# A longitudinal study of grapheme-color synesthesia in childhood: 6/7 years to 10/11 years

**DOI:** 10.3389/fnhum.2013.00603

**Published:** 2013-11-12

**Authors:** Julia Simner, Angela E. Bain

**Affiliations:** Department of Psychology, University of EdinburghEdinburgh, UK

**Keywords:** grapheme-color synesthesia, development, consistency, children, longitudinal, childhood

## Abstract

Grapheme-color synesthesia is a condition characterized by enduring and consistent associations between letter/digits and colors. This study is the continuation of longitudinal research begun by Simner et al. (2009) which aimed to explore the development of this condition in real time within a childhood population. In that earlier study we randomly sampled over 600 children and tested them aged 6/7 and 7/8 years. We identified the child synesthetes within that cohort and measured their development over 1 year, in comparison to a group of non-synesthetic children with both average and superior memories. We were able to show the beginnings of a developmental progression in which synesthetic associations (e.g., *A* = red) mature over time from relatively chaotic pairings into a system of fixed consistent associations. In the current study we return to this same population three years later when participants are now 10/11 years. We used the same paired-association memory task to determine the synesthetic status of our participants and to also establish synesthetes' inventories of grapheme-color associations. We compared their inventories to those from age 6/7 and 7/8 years to examine how synesthesia matures over time. Together with earlier findings, our study shows that grapheme-color synesthesia emerges with a protracted trajectory, with 34% of letters/digits fixed at age 6/7 years, 48% fixed at 7/8 years and 71% fixed at 10/11 years. We also show several cases where synesthesia is not developing in the same time-frame as peers, either because it has died out at an older age, or because it was slower to develop than other cases. Our study paints the first picture of the emergence of synesthesia in real-time over four years within a randomly sampled population of child synesthetes.

## Introduction

For people with grapheme-color synesthesia, letters or digits have fixed enduring conscious color associations. For example, the letter A might be red, B might be blue, C might be yellow and so on. In the terminology of the literature, letters and digits are the “inducers” of grapheme-color synesthesia, and the color itself (i.e., the synesthetic experience) is the “concurrent” (Grossenbacher, 1997). Synesthetes report that concurrent colors are either experienced in the mind's eye, or can be projected out into space onto objects such as the written typeface (Dixon et al., 2004). Studies have established that grapheme-color associations have a likely genetic aetiology (Asher et al., 2009; Tomson et al., 2011), that they are neurologically driven (e.g., Hubbard et al., 2005; Rouw and Scholte, 2007; Weiss et al., 2009) and that they tend to be automatically triggered without conscious effort on the part of the synesthete (e.g., Dixon et al., 2000). Despite a relative wealth of contemporary findings on synesthesia produced in recent years, questions about the *development* of synesthesia have been largely overlooked in empirical terms. This study aims to address this by examining how these unusual associations are acquired throughout childhood in a random sample of child grapheme-color synesthetes.

Within the field of synesthesia research, the study of development in childhood has been hindered by a difficulty in recruiting child synesthetes. Other neurodevelopmental child cohorts (e.g., children with autism) can be readily found following their referrals to clinics, but these routes are not available for researchers of synesthesia since the condition is not classified as pathology. Options for recruitment are therefore limited to inviting participation from the synesthetic children of known adult synesthetes (Green and Goswami, 2008), or to the time-intensive scanning of large random populations to detect the comparatively small number of child synesthetes within them (Simner et al., 2009). The former approach raises questions of scientific validity because the children of known adult synesthetes, often study participants themselves, are unlikely to represent the population of child-synesthetes at large. Instead they are the children of family environments led by motivated parents with an interest in scientific research and the engagement in science, and in particular with an interest in synesthesia (Simner et al., 2009). For all these reasons both the children themselves and the children's synesthesia may be non-representative of what we might expect from the average synesthete child, randomly sampled. The only study to have attempted the latter screening approach in order to find randomly sampled child synesthetes (Simner et al., 2009) was considerably time-intensive because the rarity of synesthesia requires screening large samples for comparatively small numbers of synesthetes (e.g., 8 grapheme-color synesthetes found in 615 children screened by Simner et al., 2009). However, once found, those child synesthetes were, and remain, an extremely valuable population, and we know of no other similar cohort anywhere in the world. For this reason, we continue to explore the development of these children in the current paper, showing how their condition has matured now they are four years older than when first tested.

The requirements of our current study—and those of Simner et al. (2009)—were first to have an objective means of identifying child synesthetes from a large sample of children, and second to have a way to evaluate how their synesthesia is developing over time. The test to identify synesthetes used by Simner et al. (2009), and again here, is based on the behavioral “gold standard” task (Rich et al., 2005): a test of *consistency over time*. In this test, synesthetes are identified by the fact that people with synesthesia tend to have consistent and enduring inducer-concurrent relationships. In other words, for any given synesthete, each inducer (e.g., a letter) will tend to elicit the same concurrent color over time (e.g., if the letter A is red, it tends to always be red for that particular synesthete, in repeated testing). This consistency was first demonstrated by Baron-Cohen et al. (1987) who showed that a grapheme-color synesthete reported the same colors for over 100 letters and words in two different testing sessions separated by more than two months. This consistency effect is perhaps the most replicated finding in the synesthesia literature: studies have replicated it in grapheme-color synesthesia (Dixon et al., 2000; Edquist et al., 2006; Eagleman et al., 2007), sound-color synesthesia (Ward et al., 2006), word-taste synesthesia (Ward and Simner, 2003), and so on—indeed, within virtually every study of synesthesia within the last decade. Across these studies, synesthetes tend to be between 80 and 100% internally consistent over time (e.g., Ward et al., 2005); in contrast, a non-synesthete control tends to be around 25% internally consistent when asked to generate and then recall analogous associations (e.g., to free-associate colors to letters and then recall these associations in a retest). Indeed, controls score poorly even when tested over a considerably shorter time interval (e.g., 17% consistent when retested after just 2 weeks; Baron-Cohen et al., 1987). Given this, the test of consistency has been adopted as the primary means for identifying genuine synesthesia (but see Simner, 2012 for discussion): synesthetes are required to be significantly more consistent in recalling their associations compared to a group of non-synesthete controls.

The consistency test used to identify child synesthetes in Simner et al. (2009) will be used again in the current study. Simner et al. tested 615 children aged either 6 or 7 years (which we henceforth refer to as “aged 6/7 years”). Each child took part in an on-screen test in which they viewed the letters of the alphabet and the digits 0–9, one by one, in a random order. Accompanying each grapheme was an on-screen palette of 13 colors, and children were required to pair each grapheme with a color as they saw fit. When all graphemes had been shown, the test paused for approximately 10 s, then participants repeated the test again. Simner et al. compared the grapheme-color choices made before vs. after the 10-s pause, to establish for each child how consistent his/her color choices had been across that short interval. On average, children selected the same color for only 3.5 out of the 36 graphemes. However, 47 children had consistency scores significantly higher than the mean and these were classified as “potential synesthetes”—in that their high consistency may have been indicating they were synesthetes. To further clarify this, Simner et al. returned one year later, when the children were now aged 7/8 years. Simner et al. presented the same test again, but this time compared the grapheme-color associations made by each “potential synesthete” across the entire year that had passed. By doing this they found eight children who continued to be highly consistent even across this extensive time interval. These eight children were therefore categorized as genuine synesthetes on the assumption that maintaining this consistency would be a hugely challenging task for non-synesthetes. Indeed, Simner et al. required that their synesthetes had consistent colors both across 12 months (compared to their entire peer group over just 10 s, in Session 1) and also across 10 s at age 7/8 years (and here they were compared to 40 orthogonally age/sex-matched controls who were also selected for retesting at age 7/8, because they had been representative of perfectly average non-synesthete children in Session 1). After removing these eight “genuine synesthetes,” Simner et al. classified the remaining 39 “potential synesthetes” as “high-memory non-synesthetes,” since these children had likely performed well when first tested (i.e., over 10-s) based solely on a high memory span.

In the current study we aimed to repeat this process with the same cohort of children, now that they are aged 10/11 years (i.e., 8 synesthetes, 39 high-memory non-synesthetes and 40 average-memory non-synesthetes). Our first intention was to establish whether the child synesthetes identified previously are still exhibiting the behavior of synesthetes after three more years have passed. It is possible that synesthesia may die out in some individuals, and there are three reasons to suspect this. First, there are anecdotal reports from adults suggesting they had synesthesia in childhood which they no longer have. Second, Simner et al. (2009) found a numeric trend toward finding more synesthetes in their younger (originally aged 6) vs. older (originally aged 7) age group, although the small numbers in that study gave insufficient power to test this statistically. Finally, a decline in synesthesia through childhood is also predicted by the most widely accepted developmental model of synesthesia: the *Neonatal Synesthesia Hypothesis* (Maurer, 1997). This model proposes that all neonates and young infants experience sensory/perceptual stimuli in a synesthetic manner (Spector and Maurer, 2009). However, children are thought to gradually lose these exaggerated abilities through selective pruning of synaptic connections in childhood because these connections may no longer be adaptive to development and general cognition (Spector and Maurer, 2009). Synesthetes, on the other hand, are thought to maintain these connections into adulthood, either via absent or delayed synaptic pruning. These proposals are backed up by infant neuroimaging studies: verbal stimuli can activate both verbal and visual cortex in a way that is not observed in the adult population (Neville, 1995) thereby suggesting a less modularized processing in infants. Additionally, sound and color associations have also been found in infants that mirror those observed later in older sound-color synesthetes (Mondloch and Maurer, 2004). If the neonatal synesthesia hypothesis is correct, this would suggest that synesthesia may die out in some children as the normal pruning processes in development run their course (see Huttenlocher and Dabholkar, 1997 for a discussion on the time-course of this pruning). Our study will test whether any children previously identified as synesthetes fall below that classification now that more time has passed.

As well as identifying child synesthetes, Simner et al. were able to evaluate how these children's synesthesia had developed over the one year that had passed between their two testing sessions (i.e., between Session 1 when children were 6/7 years, and Session 2 when the children were 7/8 years). Simner et al. found that on average, genuine synesthetes acquired 6.4 new fixed grapheme-color associations over that year: at age 6/7 years synesthetes were consistent over 10 s in the coloring of 10.5 graphemes on average (out of 36 letters and numbers), while at age 7/8 years they were consistent on 16.9 graphemes over 10 s. In other words, synesthetes were moving from a state where their colors were largely in flux, to a state where almost half of graphemes were fixed to a consistent color, at least over a 10-s retest. In the current study we will test how this pattern of acquisition has continued to develop now the children are aged 10/11 years. Since adults have consistent colors for around 80–100% of their graphemes, we anticipate a higher proportion of fixed colors in older vs. younger children. We will assess this by comparing how consistently our synesthetes pair graphemes with colors over a 10 s retest at 10/11 years to how they did when they were younger (at age 6/7 and /7/8 years).

In summary, Simner et al. (2009) tested children aged 6/7 years (**Session 1**) and again aged 7/8 years (**Session 2**). Children made grapheme-color selections twice in each session and we label these **Selections 1a/b** and **Selections 2a/b**. In the current study, we will test the children again in a **Session 3**, using the same task where they will again make grapheme-color selections twice across a 10-s gap (**Selections 3a/b**). We will again identify synesthetes as those children who show consistent color choices both in **immediate retest** (Selection 3a vs. 3b; i.e., a 10 s gap) and in a **delayed retest** (Selection 3 vs. Selection 1; i.e., a 4-year gap^1^). As before synesthetes will need to significantly outperform their peers on both criteria. Once synesthetes are identified here we will next evaluate how many of their graphemes now have fixed colors at age 10/11 years (Selection 3a vs. 3b) and compare this to how many were fixed at age 7/8 years (Selection 2a vs. 2b) and at age 6/7 years (Selection 1a vs. 1b). We predict that synesthetes will show an incremental increase in the number of fixed colors from the younger to the older testing age.

## Experimental investigation

The eight previously identified synesthetes and their high and average memory controls will be given the same consistency task administered in previous testing sessions by Simner et al. (2009) and will be assessed using similar criteria. The synesthetes that continue to consistently associate graphemes and colors to a significant degree, in both immediate and delayed retests, shall be re-confirmed as genuine synesthetes at 10/11 years old. The development of grapheme-color synesthesia shall then be investigated by assessing the acquisition of synesthetic associations from ages 6/7 to 10/11 in comparison to the development of non-synesthetic children with both high and average memories.

### Methods

#### Participants

We tested 80 of the 87 children previously tested by Simner et al. (2009). In that earlier study, children had been identified as either synesthete, high-memory non-synesthete or average-memory non-synesthete (see above). Seven high-memory and 10 average-memory non-synesthetes were untraceable for the current study, although we were able to replace all 10 average-memory non-synesthetes with children from the same population (i.e., children whose immediate consistency score in Session 1 fell within the average range: either 3 or 4, out of 36 graphemes). The original 40 average-memory non-synesthetes tested by Simner et al. had been orthogonally crossed for age (6 vs. 7 years in Session 1) and sex; our replacements allowed us to fully maintain this for age, and virtually maintain this for sex (19 males; 21 females). We also tested the eight children previously classified as synesthetes (five female) and the remaining 32 classified as high-memory non-synesthetes (22 females). All participants were 6/7 years when first tested by Simner et al. (2009), and are described here as aged 10/11 years for conciseness, although five children were still 9 at the time of our testing here, and ten children had just turned 12 (due to the particular timings and duration of our testing). Our study was approved by the local ethics board and written consent was given by head teachers and parents.

#### Materials and procedure

Our consistency task was a computerized test taken from Simner et al. (2009; and previously, from Simner et al., 2006) which individually presents the 26 letters and the digits 0–9 (i.e., 36 graphemes) alongside an electronic palette of 13 colors (black, dark blue, brown, dark green, gray, pink, purple, orange, red, white, light blue, light green and yellow). These colors are based on Berlin and Kay's (1969) irreducible color terms, with the addition of dark and light variants of blue and green^2^. Both the order of graphemes and the trial-by-trial configuration of colors were randomized for each participant. Graphemes were displayed in black Ariel font against a white background on the left side of the screen, taking up approximately 40% of the screen-height. As in Simner et al. (2009) participants were told they would be playing a short computer “game” and would be required to select with a computer mouse the “best” color for each grapheme as promptly as possible. They were also told that there was no right or wrong answer but that they should avoid selecting the same color repeatedly. A paper print-out of a screen-shot was used to illustrate what the task entailed. Approximately 10 s after completing all 36 graphemes, participants performed an immediate surprise retest, in which the order of graphemes and color configurations were re-randomized. (Specifically, our program presented all 36 graphemes in a random order, then paused for 10 s, then began again, showing the same, but re-randomized, graphemes. During the pause, children were told to wait a few moments and then to “carry on as before.”) The task took ~5 min to complete.

### Results

#### Synesthete status

We remind the reader that our participants have now been tested in three sessions, and that within each session they made two color selections for each grapheme, either side of a 10-s pause. We have termed these: Session 1 (age 6/7 years; **Selections 1a and 1b**), Session 2 (age 7/8 years; **Selections 2a and 2b**) and Session 3 (age 10/11 years; **Selections 3a and 3b**). Sessions 1 and 2 were conducted by Simner et al. (2009) and Session 3 is added here.

Below, we identify children who are “Session 3 synesthetes (aged 10/11 years)” by comparing how consistently our participants paired graphemes to colors both within and across testing sessions. Following Simner et al. (2009), for a child to qualify as a synesthete he/she must satisfy both of the following requirements: be significantly more consistent in *immediate consistency* (Selections 3a vs. 3b) compared to average-memory non-synesthetes AND be significantly more consistent in *delayed consistency* over four years (Selections 1a vs. 3a *or* Selections 1b vs. 3b^3^) compared to the mean consistency of their peer-group (*n* = 615) over 10 s in Session 1. (To rephrase this, *delayed consistency* requires that the associations made by synesthetes at age 6/7 years remain significantly more consistent over four years, than their peers' associations remained over 10 s only).

We shall assess all 80 children for synesthesia, notwithstanding the fact that only eight were recognized as synesthetes at an earlier age by Simner et al. (2009). This is because some true synesthetes may have evaded identification in Sessions 1 and 2, perhaps because their development was slower than their synesthetic peers. (Equally, we may also find that some cases of verified synesthesia may now have declined with age).

#### Immediate consistency

To achieve the status of a genuine synesthete at 10/11 years, a child must first significantly outperform average memory non-synesthetes (*n* = 40) tested for immediate consistency in Session 3. Participants' scores were collapsed across both age and sex since Session 3 average-memory controls showed no significant effect for either factor (although there was a trend for girls to perform better) [age: *F*_*age* (1, 36)_ < 1; *F*_*sex*(1, 36)_ = 3.31, *p* = 0.08] and there was no interaction [*F*_age*sex (1, 36)_ > 1]. We calculated a cut-off value at 1.96 standard deviations above the control mean, both for letters (mean = 4.1; *SD* = 2.9; cut-off = 9.8) and numbers (mean = 1.7; *SD* = 1.6; cut-off = 4.9). As such, synesthetes had to achieve a consistency of at least 10/26 for letters or 5/10 for numbers. We describe how participants satisfied these criteria further below.

#### Delayed consistency

The delayed consistency criterion required synesthetes to score significantly higher over a four years gap than their peer group (*n* = 615) had scored over 10 s in Session 1. In Session 1, girls had performed similarly to boys although 7 year olds (*n* = 275) significantly outperformed 6 year olds (*n* = 332). As such our delayed consistency analysis in Session 3 requires us to assess the two age groups separately. Table 1 summarizes the means, standard deviations and synesthetic criteria (1.96 SD above the mean) for each age group, from Simner et al. (2009); synesthetes who were 6 when first tested now require a 4-year delayed consistency of at least 6/26 for letters or 3/10 for numbers, while those who were 7 years require 8/26 or 4/10.

**Table 1:** **Descriptive statistics for children (*n* = 615) aged 6 and 7 years from Simner et al. (2009)**.

**Age (years)**	**Letters (/26)**			**Digits (/10)**		
	**Mean**	***SD***	**Cut-offs**	**Mean**	***SD***	**Cut-offs**
6	2.3	1.7	5.5	0.9	0.9	2.7
7	2.8	2.3	7.2	1.1	1.1	3.2

Table shows means and standard deviations (SDs) from immediate consistency over 10 s in Session 1 (i.e., Selection 1a vs. 1b) for letters and digits. It also shows the values of the “cut-offs” by which we identified synesthetes (mean plus 1.96^*^SD; i.e., synesthetes must perform significantly higher than the mean). Session 3 synesthetes must score at or above this cut-off in a four-year retest.

Six children indeed satisfied both immediate and delayed consistency criteria, and on this basis can be recognized as Session 3 synesthetes (aged 10/11 years). These were five of the eight synesthetes from Simner et al. (2009; HM, JC, DJ, GT, MA) and one child previously categorized as a high-memory non-synesthete (CM; female; aged 6 when first tested). All children who continued to be classified as such from in Simner et al. (2009) remained within the same variant(s) (i.e., either letter-color and/or digit-color) apart from DJ who moved from letter/digit-color synesthesia to just letter-color synesthesia.

We also identified six additional children who were very close to achieving our rigorous standards, three of whom were previously identified by Simner et al. (2009) as synesthetes (CC, CD, DM), two as high memory non-synesthetes (MW, ET), and one as an average memory control (MH; whom we recruited as a replacement so did not test in Session 2). These six children achieved the required consistency either for immediate or delayed consistency, and missed the other criterion by only one point (i.e., fell short on only one letter or digit). In all cases, we verified that high consistency had not been achieved by using only a limited color palette (e.g., choosing the color red throughout) and indeed, on this basis, we eliminated a further child who had also approached significance.

In Table 2 above we summaries our findings from Session 3 for all children of interest described above. Our six confirmed Session 3 synesthetes are above the triple line, and our “near-misses” (i.e., those who satisfied one criterion and almost satisfied the other) are below the line. Their statuses are shown by checks or question marks, respectively, in the final two columns. The previous status of all children at a younger age (from Simner et al., 2009) is shown by sub-script next to their ID. The table also shows immediate consistency scores for letters and digits in Session 3, with gray shading indicating cells where immediate consistency was within the synesthetic range. Note therefore, that any children considered “near misses” who do *not* have gray shading (i.e., DM and ET) satisfied synesthetic consistency in delayed, rather than immediate consistency.

**Table 2:**
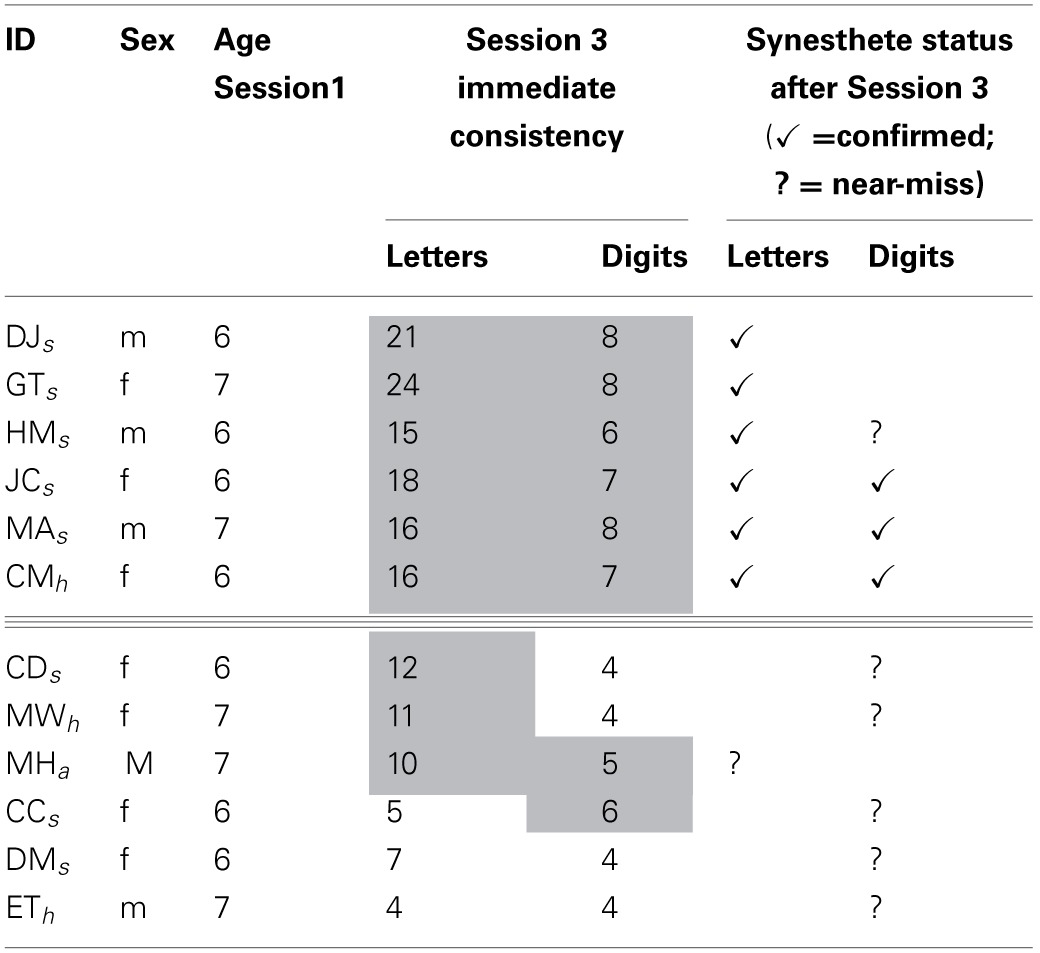
**Session 3 synesthetes and “near-misses,” indicated by checks and question marks respectively**.

Columns 1–3 show ID (with previous status in sub-script; s, synesthete, h, high-memory non-synesthete, a, average-memory non-synesthete), sex (m, male; f, female), and age in years when first tested by Simner et al. (2009). Columns 4–5 show immediate consistency in Session 3 (Selections 3a vs. 3b), out of 26 for letters, and 10 for digits. Gray highlighting indicates that this immediate consistency satisfied the synesthetic criterion.

**Figure 1:**
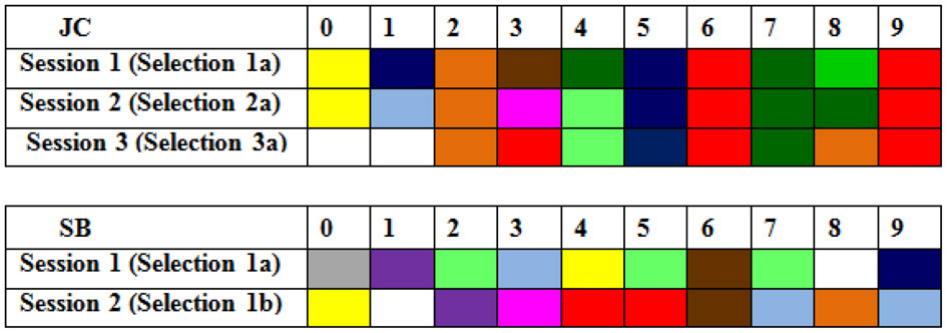
**Color choices in the first presentation of digits (0–9) in Sessions 1–3 for synesthete JC (i.e., over 4 years) and those from an aged matched control SB (over 10 s; in Session 1)**.

An example of the striking consistency of synesthetes across all three testing sessions can be seen in Figure 1. This shows the colored digits of synesthete JC when she was 6 years old, and then again one, and four years later. It is clear that the selections she made in Session 1 remain considerably more consistent over four years than those made by an age-matched average control (SB) over just 10-s.

#### Synesthetic development

Above we have identified six Session 3 synesthetes (age 10/11 years). Here we now consider the development of their synesthesia over time. By examining their immediate consistency scores in Session 3 (i.e., Selections 3a vs. 3b) we can estimate how many graphemes had their own single fixed color at this age, at least to the extent that this color remained consistent in a 10-s retest. Our approach shows that on average, 10/11 years old synesthetes had fixed colors for 25.7 out of 36 graphemes (SD 4.1), while high memory non-synesthetes had 7.4 (SD 4.4), and average non-synesthetes had 5.8 (SD 4.2). To evaluate these group differences we have remained agnostic about the status of the six “near-miss” children who fell just short of synesthesia (see above). Instead, we have left these six within their original groups (i.e., either high or average memory non-synesthetes) apart from CC, CD, and DM whom we have moved from the synesthete to high memory group (since they had superior immediate consistency in session 1 but are not currently considered synesthetes).

**Figure 2:**
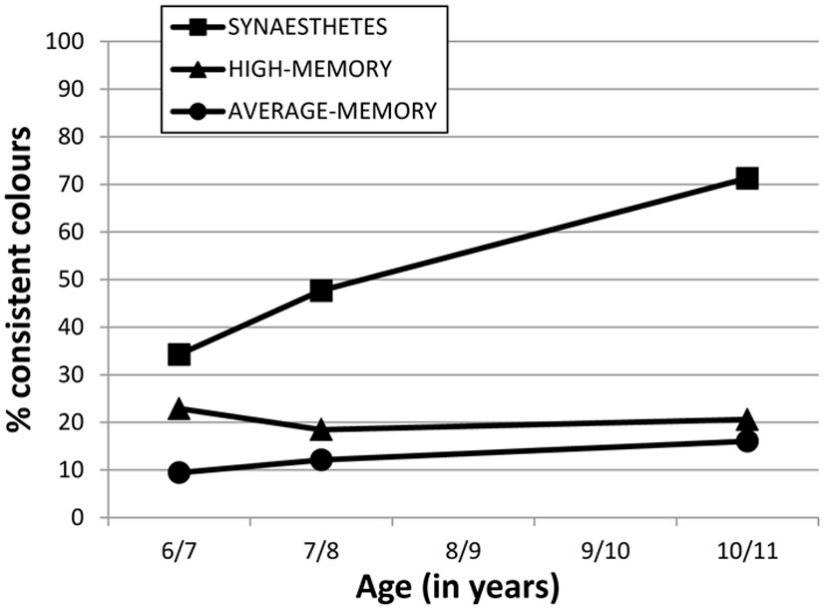
**Percentage of consistent colors selected by the three participant groups (see legend) in their immediate consistency of 36 graphemes within Sessions 1, 2 and 3 (ages 6/7, 7/8, 10/11 respectively)**. Intervening years have no data because no testing took place at these ages.

Figure 2 shows our Session 3 data combined with earlier sessions from Simner et al. (2009), to illustrate how synesthetes have developed fixed colors over time. Figure 2 shows the immediate consistency for 36 colored graphemes (over 10-s) at ages of 6/7 years (Selections 1a vs. 1b) and 7/8 years (Selections 2a vs. 2b) and 10/11 years (Selections 3a vs. 3b). We have converted these consistencies to percentages to better illustrate the rate of development.

Figure 2 shows that synesthetes have a sharper rate of acquisition of colored graphemes, compared to what might be expected from other children with average or high-memories. Over the 4-year testing period, synesthetes acquired on average 13.3 new fixed colored graphemes (9.2 letters, and 4.0 digits, within each variant), while high and average memory non-synesthetes improved across sessions by less than 3 graphemes.

**Figure 3:**
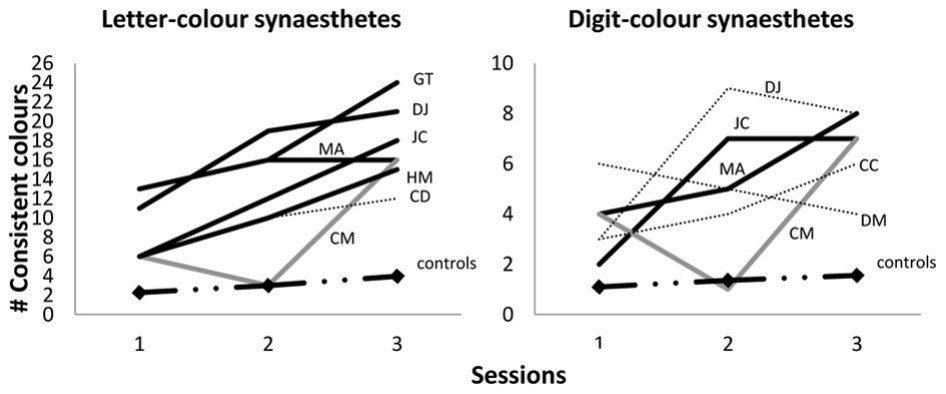
**Number of consistent colors selected within Sessions 1, 2 and 3 (ages 6/7, 7/8, 10/11 respectively) for letter-color synesthetes (out of 26; left panel) and digit-color synesthetes (out of 10; right panel)**. Black lines indicate Session 3 and 2 synesthetes; gray lines indicate Session 3 (only) synesthetes; dotted lines indicate Session 2 (only) synesthetes; dash/dotted lines with diamond data-markers indicate the means of average memory controls.

We point out that that a subset of our synesthetes achieved synesthetic status for letters only (rather than for all graphemes) meaning that they should not be expected to achieve 36 fixed colored graphemes even in a fully-formed adult state. However, we can report that the pattern of acquisition shown in Figure 2 is a good approximation for the pattern when synesthetes are broken down by type. For example, Figure 2 shows approximately 71.3% of the 36 graphemes were fixed in color for all synesthetes at aged 10/11 years, and indeed this figure is very similar for those with the letter-color variant (70.5%) or the digit-color variant (73.5%). Nonetheless, for further clarification on how synesthesia develops within each variant, Figure 3 below shows the case-by-case development of letter-color synesthetes (left panel) and digit-color synesthetes (right panel) separately. We have included not only children classified as synesthetes in both Session 3 and Session 2 (indicated by solid lines), but also those classified in Session 2 only (indicated by dotted lines) and those classified in Session 3 only (indicated by gray, rather than black, lines). These latter two groups can be thought of as children having synesthesia that is, respectively, either dying out, or developing more slowly than their peers. Finally, we also show in Figure 3 the progression of average memory controls (dash/dotted line with diamond data-markers).

### Discussion

Our study re-assessed the synesthetic status of children aged 10/11 years who had previously been identified as synesthetes by Simner et al. (2009) when they were aged 6/7 and 7/8 years. We again identified synesthetes using the gold standard behavioral measure assessing how consistently graphemes are paired with colors over time. In our test, children aged 10/11 years paired graphemes with colors, and their selections were compared across a 10-s retest (*immediate consistency*), and also to selections they had made four years earlier (*delayed consistency*). Like Simner et al. (2009) we required synesthetes to outperform controls in both types of measure. We also assessed the synesthetic status of a group of children who had previously been classified as being non-synesthetes with high memories, on the assumption that some may in fact be more-slowly developing synesthetes. Finally, we also tested here a group of average memory non-synesthetes to serve in a control condition. Once we had identified who were synesthetes from among our total sample, we compared their inventories of colored graphemes at different ages to observe how their synesthesia had developed over time (from age 6/7 to 7/8 to 10/11 years).

We found that five of the eight children previously identified as grapheme-color synesthetes at a younger age continued to satisfy synesthetic criteria in our current study. In other words, these children have now shown hallmarks of synesthesia across four years. We also found a sixth synesthete, not previously classified as such in earlier testing. Her case is unusual: her colors were consistent over 10 s when she was 6, then over one year, and again over 4 years, and they were also highly consistent over 10-s within this final test session (aged 10/11). However, in Session 2 there was a lot of variation in her colors when immediately retested, and for this reason she was classified by Simner et al. (2009) as a high-memory non-synesthete. If our current assumption is correct, and she is indeed a genuine synesthete, we have two possible interpretations: either there was a period of particular flux in her synesthetic colors when she was 7 years old (in Session 2) or she was tired/distracted during that test. The latter is at least plausible, since her first selection of colors in Session 2 (i.e., Selection 2a) were significantly consistent with those from a younger age, while her second selection (Selection 2b) was not (0% match). It is possible therefore that her attention to the task waned during the second testing session conducted by Simner et al. (2009).

Our study might also lend support to the notion that synesthetic ability can die out over time, because three children who were previously classified as synesthetes at an earlier age no longer satisfied these requirements in our most recent test. Since each evaluation of synesthesia is made against age-matched controls, we can assume these children *were* synesthetes at an earlier testing session, and hence, we might therefore have empirical evidence of what could be considered “synesthetic demise” (i.e., the decline of synesthesia within an individual such that it is no longer apparent at a recognized level at a later age). This seems to fit with previous anecdotal evidence from adults who have given subjective accounts of synesthetic ability in childhood that they no longer possess as adults. This is also evidenced by the fact that one of our Session 3 synesthetes has gone through a transition from being a letter and digit synesthete at age 7/8 to only a letter synesthete at age 10/11. Nonetheless, it is possible too that the synesthesia of these three children is merely different in characteristic to those of their peers. Two of these cases in particular were highly consistent over 10 s when tested here, but failed to show high enough consistency compared to the colors they provided four years ago. It is therefore possible that they remain synesthetes but that their colors have simply developed away from those first experienced when they were much younger. If this is true, we would predict that a future test of consistency will show high consistency between their current inventories (age 10/11), and those generated at a future date.

For our unequivocal synesthetes, we also examined their development across four years and found that grapheme-color synesthesia appears to moves from a more chaotic to less chaotic system. When synesthetes were 6/7 years old, only around a third of graphemes on average had colors fixed enough to endure in a 10-s retest. By the age of 7/8 this had risen to almost 50%, and at age 10/11 this is now over 70%. This development far exceeds the type of normal age-related changes we might expect from non-synesthetic children in the same task, as evidenced by the performance of our average- and high-memory controls (see Figure 2). Our study also allows us to confirm that the rate of acquiring fixed colors for letters slows down as synesthetic children age (see Figure 2). Synesthetes acquired on average 6.4 graphemes over the 12 months between Sessions 1 and 2, but only another 4.2 graphemes across the entire three years that followed. A more linear acquisition would have predicted that synesthetes would have acquired all 36 grapheme-color associations by age 10/11 years. Instead, we found that synesthetes are still below the 100% consistency that can characterize fully mature grapheme-color synesthesia, although our children are developing at different rates. Despite a the group-mean of ~70%, synesthete MA already has 80% of his digits fixed with color, while GT has 92% of her letters. Hence although it may take several more years before these children achieve their full complement of grapheme-color associations, some are likely to achieve this sooner than others. It remains a point of investigation to track these children into stable adult-like profiles before we can appreciate the complete picture of the development of grapheme-color synesthesia.

This third installment of findings on the development of grapheme-color synesthesia provides valuable insights because it shows how synesthesia unfolds over time. Another recent study has also provided compatible data on development, this time in a far younger population. Spector and Maurer (unpublished, reported in Maurer et al., 2013) conducted a longitudinal study testing three pre-school children who were aged 3.5–4.5 years when the test began. These children had synesthetic mothers and so were reasonably likely to be synesthetes themselves, given familial inheritance patterns (e.g., Ward and Simner, 2005). Spector and Maurer provided 96 colored crayons, and children colored up to one grapheme per day and then immediately repeated the cycle, between three and six times. These three putative synesthetes were far more consistent on their choice of colors across cycles than age-matched controls (who were not at all consistent), and their consistency increased as the cycles advanced; children were 40% consistent in the first two cycles, aged 3.75–4.75, but were around 75% consistent in the final two cycles, age 4.5–5.5. This very high consistency score at age only 5.5 years, in the face of our own data, suggests these may be somewhat special synesthetes. Our own study here of randomly sampled child synesthetes predicts that consistency around age 5 should be less than 30%. We see two possible interpretations: either this study happened to sample particularly synesthetic children, or the task itself—with its repetitive coloring of graphemes performed on between 78 and 156 different days depending on the child—may have reinforced colors earlier than otherwise expected. A final possible interpretation is that the children self-referred by research-interested synesthetic parents may be non-representative of more randomly sampled children *per se*. What is interesting, however, is that both this study and our own shows the development of synesthesia from more fluctuating to less fluctuating colors.

The issue of self-referral is an important one, on which we will therefore dwell a little longer. Other studies have also investigated childhood synesthesia, but again used samples of children likely to have been brought forward for testing by their synesthetic parents. Green and Goswami (2008) showed that a synesthetic population of children show cognitive benefits (e.g., in vocabulary and mathematics). Although Green and Goswami provided valuable information about the cognitive abilities of these children, it is not clear how much their findings can be extended to child synesthetes in general. It is likely that the children of parents with an interest in science, and the engagement in science, and in synesthesia in general, may have children who naturally score higher in the types of tests used in this study in any case. Hence, we are currently testing our own population, of randomly sampled synesthetes, on a range of test of cognitive tests in the hope this will reveal the true abilities of child synesthetes randomly sampled.

Although we have successfully identified a number of child synesthetes and tracked their development, we must acknowledge the shortfalls of our methodology based on the same reasons identified by Simner et al. (2009). In particular, we point out that our methods will necessarily underestimate the prevalence of childhood synesthesia, and this is because it relies on the test of consistency over time. Although this is a reliable test for adult synesthetes, who are generally highly consistent (e.g., 100%) in their synesthetic associations, our study itself shows that child synesthetes only acquire this consistency with time. Hence any study aiming to identify child synesthetes by their consistency is using a necessarily weak metric (see Simner et al., 2009 for full discussion). Furthermore, recent papers (Cohen Kadosh and Terhune, 2012; Eagleman, 2012; Simner, 2012) have asked whether high consistency is a true hallmark of synesthesia at all, or whether instead it characterizes only a subset of synesthetes. In other words, although all contemporary studies identify synesthetes by their consistency over time there may yet be some synesthetes overlooked by these studies who have varying and fluctuating colors, even into adulthood. Given both of these considerations, our study may have identified only a subset of child synesthetes with particularly early-developing synesthesia, or with particularly consistent synesthesia. Nonetheless, our study remains informative about this particular population *per se*.

Our study might be considered alongside theories about neurodevelopment in synesthesia. We have seen that grapheme-color synesthesia already exists in a nascent state at age 6 years, and that it develops slowly through childhood. Studies of adult grapheme-color synesthetes suggests it arises through “cross-activation” between adjacent brain regions involved, on the one hand, in the recognition of graphemes and words (the “visual word form area”; VWFA) and on the other, in color processing (e.g., V4; Hubbard et al., 2005; Rouw and Scholte, 2007). Importantly, since grapheme-color synesthesia relies on knowledge of culturally acquired symbols, it could not exist as such in in infancy. Hence, although the left-hemisphere language areas are already well-organized for the perception of spoken language in infants (Dehaene-Lambertz et al., 2006a,b) it is only later, when learning letters and numerals, that the types of representations required to drive grapheme-color synesthesia can be established. Here we see grapheme-color synesthesia developing in behavioral terms, alongside these children's growing familiarity with letters and digits.

In summary, we have identified child synesthetes, and observed their development longitudinally over three testing sessions spanning four years. We have seen that their synesthesia develops from relatively fluid pairings of graphemes to colors, which form more fixed associations at later ages. We have seen cases of synesthesia perhaps dying out over time, or developing more slowly in some individuals over others. We have been able to say with relative certainty that six children in our sample are synesthetes, and that a further six may be potential cases to be observed in future studies. Indeed, a number of questions raised here can be addressed by future testing, and we have one such study in preparation. Our participants were 10/11 years old at the time of testing for the current paper but are now 14/15 years of age. We therefore anticipate that their status as synesthetes might now be further informed by their own meta-awareness. Older synesthetes are able to self-report whether they have synesthetic experiences in a way that is far less tractable in younger children (and hence self-disclosure of synesthesia was not used as part of the methodology in the current study). Future observations of these synesthetes into adulthood might therefore rely on both objective and subjective reporting. Moreover, future studies might ask whether or to what extent similar developmental trajectories also characterize other forms of synesthesia, such as colors triggered by sound/music for example. Sound-color synesthesia relies on different “cultural artifacts,” such as cultural dictates for musical meter and pitch structure, characteristics which are learned even in infancy (Soley and Hannon, 2010). Such differences in development with respect to inducers might therefore be expected to cause different developmental trajectories in the synesthesia itself. We hope that future studies might find ways to overcome existing obstacles in recruiting of child synesthetes, which have thus far hindered developmental research in the field. We also anticipate that future studies could provide information about the implications of synesthesia on other aspects of cognitive development. Green and Goswami (2008) suggest that young synesthetes might have a particular cognitive profile different from their peers. In addition, Simner et al. (2009) showed that synesthesia is relatively common in schools, with on average two to five grapheme-color synesthetes in every primary schools in the United Kingdom and United States respectively. If synesthesia can be linked to a particular cognitive profile (i.e., assets or deficits), as current adult studies *also* suggest (e.g., Meier and Rothen, 2013) then it will become more important to identify and understand synesthesia in childhood, and to address the particular educational needs of synesthetic children.

### Conflict of interest statement

The authors declare that the research was conducted in the absence of any commercial or financial relationships that could be construed as a potential conflict of interest.

## Footnotes

^1^See methods for how delayed consistency is calculated with respect to the four different comparisons possible here (Selections 3a vs. 1a; 3b vs. 1b; 3a vs. 1b; 3b vs. 1a).

^2^Simner et al. (2009; p. 59) point out “[a]lthough synesthetes” colors are highly specific (e.g. Ward et al., 2005) and could be closely approximated with sufficient time and effort using an extensive palette comprising many thousands of colors (as we have done elsewhere; e.g., Simner et al., 2006), prior studies have shown that a presentation of our condensed palette allows for a successful assessment of synesthesia that is practical for large numbers of participants, or for those with limited attention (Day, 2005; Simner et al., 2006). When faced with the core palette, synesthetes are systematic in their choices, while non-synesthetes are significantly more random (Simner et al., 2006).”

^3^Following Simner et al. (2009), we point out that children gave four colors for each grapheme in total in Sessions 1 and 3. Therefore, delayed consistency could, in theory, be calculated by any of four different comparisons (Selections 1a vs. 3a; 1b vs. 3b; 1a vs. 3b; 1b vs. 3a). As in Simner et al. (2009), we conservatively discounted matches across first and second presentations (e.g., Selections 1a vs. 2b), and conservatively disallowed high consistency to be achieved by a mix of consistency from within first presentations and within second presentations (e.g., four consistently cultured graphemes in Selections 1a vs. 2a, and three consistently cultured graphemes in Selections 1b vs. 2b does not equal a score of seven). For further conservativeness, we discounted any matches across light and dark versions of the same color (e.g., “light green” vs. “dark green” was considered a mismatch).
